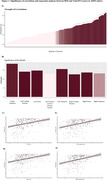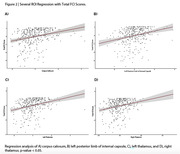# Associations between brain metabolism and financial capacity in Alzheimer’s disease

**DOI:** 10.1002/alz.093143

**Published:** 2025-01-03

**Authors:** Luca Marie Michel, Wyllians Vendramini Borelli, Thomas Hugentobler Schlickmann, Sarah Wehle Gehres, Marco De Bastiani, Andrei Bieger, Guilherme Povala, Luiza Santos Machado, Eduardo R. Zimmer

**Affiliations:** ^1^ Universidade Federal do Rio Grande do Sul, Porto Alegre, Rio Grande do Sul Brazil; ^2^ University of Applied Science Hamm‐Lippstadt, Hamm, Nordrhein‐Westfalen Germany; ^3^ Memory Center, Hospital Moinhos de Vento, Porto Alegre, RS Brazil; ^4^ Federal University of Rio Grande do Sul, Porto Alegre, Rio Grande do Sul Brazil; ^5^ University of Pittsburgh, Pittsburgh, PA USA; ^6^ McGill University, Montreal, QC Canada; ^7^ Brain Institute of Rio Grande do Sul ‐ Pontifícia Universidade Católica do Rio Grande do Sul, Porto Alegre, Rio Grande do Sul Brazil

## Abstract

**Background:**

Individuals with early stages of cognitive decline face a significant stagnation in their financial capacity, leading to a decrease in quality of life. However, whether changes in brain function are associated with financial capacity remains unclear. Here, we evaluate the association between financial capacity and brain glucose metabolism.

**Method:**

Individuals across the Alzheimer’s disease (AD) clinical continuum with complete FCI‐SF total scores and FDG‐PET imaging data were selected from the ADNI dataset. We performed a Pearson correlation with 79 brain regions of interest, extracted using the ICBM152 atlas, and the total FCI score. A multiple linear regression model was fitted to assess the relationship between FCI and different brain regions, corrected for age, gender, and education. Analyses were adjusted by Bonferroni, with p considered significant if < 0.05.

**Result:**

A total of 1364 individuals were analyzed in this study (44% female, mean age 73.4). There were 26 brain areas in which glucose metabolism significantly correlated with FCI scores (adjusted p < 0.05, Figure 1A). The highest correlation coefficients were bilaterally located in the Caudate Nucleus and Fornix (r > 0.3, Figure 1A). The regression model demonstrated multiple significant brain regions (adjusted p < 0.05, Figure 1B). The left Fornix (β = 14.8, Figure 1C) and right Fornix (β = 13.7, Figure 1D), as well as the left Caudate Nucleus (β = 14.15, Figure 1E) and right Caudate Nucleus (β = 12.66, Figure 1F), had the highest significance (adjusted p < 0.001).

**Conclusion:**

We observed positive associations between regional brain glucose metabolism and the FCI‐Score. Thus, our findings suggest a relationship between metabolic patterns, serving as an index of brain function, and the financial capacity of individuals.